# Immortalized Dorsal Root Ganglion Neuron Cell Lines

**DOI:** 10.3389/fncel.2020.00184

**Published:** 2020-06-19

**Authors:** Rainer Viktor Haberberger, Christine Barry, Dusan Matusica

**Affiliations:** Anatomy & Histology, College of Medicine and Public Health, Flinders Health & Medical Research Institute, Flinders University, Adelaide, SA, Australia

**Keywords:** DRG, immortalized cell lines, 50B11, F-11, ND7/23, ND-C, Med17.11, HD10.6

## Abstract

Pain is one of the most significant causes of suffering and disability world-wide, and arguably the most burdensome global health challenge. The growing number of patients suffering from chronic pain conditions such as fibromyalgia, complex regional pain syndrome, migraine and irritable bowel syndrome, not only reflect the complexity and heterogeneity of pain types, but also our lack of understanding of the underlying mechanisms. Sensory neurons within the dorsal root ganglia (DRG) have emerged as viable targets for effective chronic pain therapy. However, DRG’s contain different classes of primary sensory neurons including pain-associated nociceptive neurons, non-nociceptive temperature sensing, mechanosensory and chemoreceptive neurons, as well as multiple types of immune and endothelial cells. This cell-population heterogeneity makes investigations of individual subgroups of DRG neurons, such as nociceptors, difficult. In attempts to overcome some of these difficulties, a limited number of immortalized DRG-derived cell lines have been generated over the past few decades. *In vitro* experiments using DRG-derived cell lines have been useful in understanding sensory neuron function. In addition to retaining phenotypic similarities to primary cultured DRG neurons, these cells offer greater suitability for high throughput assays due to ease of culture, maintenance, growth efficiency and cost-effectiveness. For accurate interpretation and translation of results it is critical, however, that phenotypic similarities and differences of DRG-derived cells lines are methodically compared to native neurons. Published reports to date show notable variability in how these DRG-derived cells are maintained and differentiated. Understanding the cellular and molecular differences stemming from different culture methods, is essential to validate past and future experiments, and enable these cells to be used to their full potential. This review describes currently available DRG-derived cell lines, their known sensory and nociceptor specific molecular profiles, and summarize their morphological features related to differentiation and neurite outgrowth.

## Introduction

Cell bodies of sensory neurons are situated in dorsal root ganglia (DRG) or trigeminal ganglia (TG) and possess pseudounipolar processes that divide into two axons, one to innervate targets in the periphery, and the other projecting centrally in the spinal cord dorsal horn (DRG), or in the case of the trigeminal sensory nuclear complex, in the brain stem (TG). These sensory neurons, expressing an abundance of channels and receptors, monitor the environment and convey information including nociception, temperature and mechanosensation to the central nervous system (CNS) (Platika et al., 1985b; Wood et al., 1990). A substantial proportion of pain research focusses nociceptors, the subgroup of neurons involved in nociception and pain signaling, increasing the understanding of their basic functional properties and their critical roles in the development and maintenance of chronic pain (Wood et al., 1990; Rugiero and Wood, 2009), as well as identifying potential targets for therapeutic intervention. In addition to nociceptors, DRG also contain cell bodies of non-nociceptive temperature sensing, mechanosensory and chemoreceptive neurons, as well as multiple non-neuronal cell types such as immune cells and endothelial cells. The heterogeneous population of sensory neurons within the DRG can be subdivided in multiple subpopulations based on morphological, neurochemical, electrophysiological and transcriptional characteristics (Platika et al., 1985b; Wood et al., 1990; Rugiero and Wood, 2009). This cell-type heterogeneity creates a complex cellular and molecular environment making the investigation of signaling pathways related to specific classes of neuron extremely challenging. For example, analysis of total RNA or proteins obtained from DRG explants (Raymon et al., 1999) reflects average RNA expression levels across multiple groups of neuronal and non-neuronal cells rather than expression levels in a subpopulation of cells such as nociceptive neurons, or a specific type of nociceptor.

Dissociation of DRG neurons either by methods such as fluorescence assisted cell sorting (FACS) (Vetter and Lewis, 2010), or magnetic beads can isolate certain cell types but these methods produce low neuronal yields, require a high number of laboratory animals for isolation of samples suitable for proteomic or biochemical analysis, and are difficult to further it remains challenging to adequately separate subpopulations of cells. The characteristics of small numbers of specific neurons can be assessed only by a limited number of methods such as single cell RT-PCR. Investigations of signaling pathways are compromised by the isolation process and factors such as enzymatic digestion and mechanical stress. In summary, the existence of multiple types of nociceptors coupled with technical limitations in isolating individual nociceptor subtypes from native tissues have limited the advance of knowledge regarding the precise function of specific nociceptors.

Complementing *in vitro* models of isolated and dissociated DRG neurons, immortalized cell lines despite their limitations, have enabled important advances in understanding sensory neuron function. They retain significant phenotypic similarities to primary cultures in addition to greater suitability for high throughput assays due to high consistency, reducing the number of experimental animals required as well as reducing other costs normally associated with isolation and dissociation of primary cells. In the last 3 decades only a handful of DRG-derived immortalized sensory neuronal cell lines have been generated. These include the hybrid cell lines F-11, ND-C, and ND7/23 (Platika et al., 1985b; Wood et al., 1990; Rugiero and Wood, 2009), the mouse cell line MED17.11, the rat cell line 50B11 and human HD10.6 cells (Raymon et al., 1999). All of these cellular models of sensory DRG neurons that were inherently valuable for initial investigations of signaling pathways and responses to drugs and toxins. As expected, each of the aforementioned cell lines has its own unique nociceptor-like characteristics. Hence, a comprehensive evaluation of their advantages and disadvantages when compared directly to naïve sensory DRG neuron subtypes would be helpful to the broader research community. In this review we aim to provide an objective comparison of different elements encompassing DRG derived cell line experiments. This review will provide a platform to compare culture conditions for each cell line, highlighting how have these changed over the years, the methods that were used to investigate the cells and importantly what receptors, signaling pathways and functions have been identified in each cell line. In addition, each cell line will be described regarding its characteristics and usefulness for specific techniques. Particular attention will also be given to describing the use of DRG cell lines to investigate nociceptive signaling.

Immortalized sensory neuron cell lines present valuable tools for the investigation of many aspect of neurobiology, including nociception. All of the cell lines created thus far share a multitude of characteristics with both embryonic and adult mammalian DRG neurones. However, as is the case with most cell lines, these are also contrasted with major characteristic differences that need to be carefully considered prior to their experimental selection. Often these cells do not recapitulate specific DRG neuron subtypes, such as peptidergic and non-peptidergic nociceptors and analysis of calcium responses to nociceptor-related agonists and RNAseq studies have demonstrated marked differences between available DRG-derived cell lines and naïve, primary cultured DRG neurons (Yin et al., 2016; Vetter and Lewis, 2010).

A major factor that explains differences in expression profiles and function is the fact that DRG sensory neurons used in the generation of cell lines are usually of embryonal or neonatal origin. Immortalized neuronal cells generally require exposure to growth factors such as nerve growth factor (NGF) and glial-derived growth factor (GDNF), or high intracellular levels of cAMP induced via dibutyryl cAMP (db-cAMP), activated by agents such as forskolin or retinoic acid (Ghyselinck and Duester, 2019) to differentiate into a postmitotic neuronal phenotype. This phenotype is evident by reduced or absent cell division and the formation of neurites, often accompanied by changes in cell body morphology, and expression levels of molecules characteristic of adult sensory neurons (Boland and Dingledine, 1990a, b).

Recognizing the distinct functional, morphological and neurochemical characteristics of each immortalized sensory cell line compared to native neurons enables judicious interpretation of experimental results, so that these tools can continue to enable important advances in sensory neurobiology. Here we describe each individual cell line including its origin, neurochemical characteristics including sensory and nociceptor-related molecules and endogenous ion channels, and features related to differentiation and neurite outgrowth presented in tables that summarize different methodologies in relation to culturing and differentiating these cells.

### F-11 Cells

The immortalized DRG cell line F-11 was generated and first described by Platika et al. (1985a; 1985b). Sensory neurons, isolated from rat DRG at embryonic days 13–14 were fused with the mouse neuroblastoma cell line N18TG2 via exposure to 55% polyethylenglycol for 2 min. The somatic hybrids were initially grown in Ham’s F-12 medium with the addition of hypoxanthine, aminopterin and thymidine (HAT) (Tables 1, 3) to eliminate the parent neuroblastoma N18TG2 which does not express hypoxanthine phosphoribosyltransferase (HPRT).

**TABLE 1:** Maintenance and differentiation media for dorsal root ganglion cell lines.

Name and original description	Maintenance media		Differentiation media	
F11 Platika et al., 1985a, b	Platika et al. (1985b) Ham’s F-12, 15% FCS, HAT1, No Pen/Strep. Francel et al. (1987a) Ham’s F-12, 20% FCS, HAT2, 50 IU Penicillin, ? Streptomycin. Boland and Dingledine (1990a) Ham’s F-12, 15–20% FCS. HAT2, 10 mM glucose, 12 mM sodium-bicarbonate, no Pen/Strep. Linnik et al. (1993) Ham’s F-12, 20% FCS, HAT2, 50 m/ml Penicillin, 50 mg/ml Streptomycin, 50 mg/ml Neomycin.	Kim et al. (2003) DMEM, 10% FBS, 100 IU Penicillin, 100 mg/ml Streptomycin. Rimmerman et al. (2008) Ham’s F-12, 17% FCS, ? HAT, ? L-glutamine. Vetter and Lewis (2010) Ham’s F-12, 10% FCS, HAT2. Ambrosino et al. (2013) DMEM, 10% FBS, 2 mM L-glutamine, 100 IU Penicillin,? Streptomycin.	Francel et al. (1987a) Ham’s F-12, 1% FCS, 0.5 mM db-cAMP, 50 ng/ml NGF (mouse saliva), 2 mM retinoic acid, 5 mg/ml insulin, 100 mg/ml transferrin, 10 mM IBMX, 50 IU Penicillin, ? Streptomycin. Boland and Dingledine (1990a) Ham’s F-12, 1% FCS, HAT2, 0.5 mM db-cAMP, 50 ng/ml NGF (2.5S), 10 mM IBMX, no Pen/Strep. Boland and Dingledine (1990b), Ham’s F-12, 1% FCS, HAT2, 0.5 mM db-cAMP, 50 ng/ml NGF (2.5S), 10 mM IBMX; second experiment. 2 mM retinoic acid, 5 mg/ml insulin (bovine), 10 mg/ml transferrin, no Pen/Strep. Martinez et al. (2019) DMEM (no sodium pyruvate). *Three media* – *first* 0.5% FCS, 1 mM db-cAMP, 30 μM forskolin. *Second*: 0.5% FCS, 0.5 mM db-cAMP, 2 ng/ml NGF. *Third*: 0.5% FCS, 10 mM retinoic acid.	Martin et al. (2002) Ham’s F-12, 2 mM glutamine. 15% HAT, 100 U/ml Penicillin, 100 μg/ml Streptomycin, 0.5 mM db-cAMP, 10 ng/ml NGF. Kim et al. (2003) DMEM, 0.5% FBS, 0.5 mM db-cAMP OR 30 μM forskolin. Hwang et al. (2008) DMEM, 0.5% FBS, 0.5 mM db-cAMP. Vetter and Lewis (2010) DMEM, 0.5% FBS, 1 mM db-cAMP, 2 nM NGF. Xu et al. (2010) DMEM, 0.5% FBS, 10 mM forskolin, 1% Pen/Strep. Gall-Ianotto et al. (2012), Keratinocyte-serum-free-media (K-SFM), 10 ng/ml NGF, 0.5 mM db-cAMP. Ambrosino et al. (2013), DMEM, 2% FBS, 10 mM retinoic acid. Hashemian et al. (2017) Ham’s F-12, 0.5% FCS, 1 mM db-cAMP, 200 mg/ml NGF, 100 IU Penicillin, 100 mg/ml Streptomycin. Naruse et al. (1992) Ham’s F-12, 1% FCS, 0.5 mM db-cAMP, crude NGF, 2 mM retinoic acid, 5 mg/ml insulin (bovine), 100 mg/ml transferrin, 10 mM IBMX, 50 IU Penicillin, ? Streptomycin. Pastori et al. (2019) DMEM, 1% FBS, 2 mM glutamine, Penicillin?, Streptomycin?.
**ND7/23** Wood et al., 1990	Wood et al. (1990) Leibowitz L-15, 10% FCS, 3.3 g/l NaHCO3, 3 g/l glucose, 100 U/ml Penicillin, 100 mg/m, Streptomycin. Dunn et al. (1991) Leibowitz L-15, 10% FCS. Roobol et al. (1995) DMEM, 10% FBS, 2 mM glutamine, 100 U/ml Penicillin, 100 mg/ml Streptomycin. Wu et al. (2008) DMEM, 10% FBS, 2 mM glutamine, 50 U/ml Penicillin, 50 mg/ml Streptomycin.	Leffler et al. (2012) DMEM, 10% FBS, 25 mM HEPES, 3 mM taurine, 100 U/ml Penicillin, ? mg/ml Streptomycin. Milton (2012) DMEM/Ham’s F-12 mix, 10% FCS, 1% non-essential amino acids, 100 U/ml Penicillin, 100 mg/ml Streptomycin. Takaku and Sango (2020) DMEM/Ham’s F-12 mix, 5% FCS, ? U/ml Penicillin, ? mg/ml Streptomycin. Ma et al. (2020) DMEM, 10% FBS, 1% Penicillin, 1% Streptomycin, 0.11 g/L sodium-pyruvate.	Wood et al. (1990) L-15, 0.5% FCS, 1 mM db-cAMP, 2 mg/ml NGF. Dunn et al. (1991) L-15, 0.5% FCS, 1 mM db-cAMP, 2 ng/ml NGF. Roobol et al. (1995) DMEM, 0.5% FBS, 2 mM glutamine, 1 mM db-cAMP, 200 ng/ml NGF, 100 U/ml Penicillin, 100 mg/ml Streptomycin. Sivasubramaniam et al. (1997) DMEM, 10% FCS, 0.03% glutamine, 1 mM retinoic acid. Wu et al. (2008) DMEM, 0.5% FBS, 2 mM glutamine, 1 mM db-cAMP. OR 100 ng/ml NGF. Ma et al. (2020) DMEM, 10% FBS, 1 mM db-cAMP, 10 ng/ml NGF, 1% Penicillin, 1% Streptomycin, 0.11 g/L sodium-pyruvate.	Milton (2012) DMEM/Ham’s F-12 mix, 10 μM forskolin, 100 mM IBMX. Zhang et al. (2017, 2019) DMEM/Ham’s F-12 mix, 0.5% FBS, 1 mM db-cAMP, 50 ng/ml NGF, 20 mM uridine, 20 mM fluorodeoxyuridine. Martinez et al. (2019) DMEM (no sodium pyruvate). *Three media – first:* 0.5% FCS, 1 mM db-cAMP, 60 μM forskolin, 2 ng/ml NGF. *Second:* 0.5% FCS, 1 mM db-cAMP, 1.3 ng/ml NGF. *Third:* 2% FCS, 20 mM retinoic acid, 100 mM IBMX, 2 ng/ml NGF.
50B11 Chen et al., 2007	Chen et al. (2007) Neurobasal medium, 10% FBS, 0.5 mM glutamine, 0.2% glucose, 1 × B-27 supplement. Van Opdenbosch et al. (2012) Neurobasal medium, 10% FCS, 0.27% glutamine, ? glucose (20%), 2% B-27 supplement, 0.1% blasticidin. Pundavela et al. (2014) Neurobasal medium, 10% FCS. Gnavi et al. (2015) Neurobasal medium, 10% FCS, 2% B-27 supplement, 0.22% glucose, 0.2 mM glutamine.	Bestall et al. (2018) Neurobasal medium, 10% FCS, ? B-27 supplement, 0.55 mM glutamine, 11 mM (0.2%) glucose. Mohiuddin et al. (2019) Neurobasal medium, 5% FCS, ? B-27 supplement, ? glutamine.	Chen et al. (2007) Neurobasal medium, 10% FBS, 0.5 mM glutamine, 0.2% glucose, 1 × B-27 supplement, 50 μM forskolin. *In some experiments.* 10 ng/ml NGF, 10 ng/ml GDNF. Vetter and Lewis (2010) Neurobasal medium, 75 μM forskolin. Bhattacherjee et al. (2014) Neurobasal medium, 75 μM forskolin *After 17 h* 50 ng/ml NGF, 50 ng/ml GDNF, 20 nM estrogen, 100 nM angiotensin II. Pundavela et al. (2014) Neurobasal medium, 10% FCS, 5 mM forskolin.	Hulse et al. (2015) Neurobasal medium, 10% FCS, ? B-27 supplement, 0.55 mM glutamine, 11 mM (0.2%) glucose. *After 48 h* 75 μM forskolin. Bestall et al. (2018) Neurobasal medium, 10% FCS, ? B-27 supplement, 0.55 mM glutamine, 11 mM (0.2%) glucose, 1 nM NGF, 75 μM forskolin. Mohiuddin et al. (2019) Neurobasal medium, 5% FCS, ? B-27 supplement, ? mM glutamine, 10 mM forskolin. Gnavi et al. (2015) Neurobasal medium, 10% FCS, 2% B-27 supplement, 0.22% glucose, 0.2 mM glutamine. *After 24 h* 75 μM forskolin.
MED17.11 Doran et al., 2015	Doran et al. (2015) DMEM/Ham’s F-12 mix, 10% FBS, ? glutamate, 50 U/ml IFNγ, 0.5% chick embryonic extract, ? Penicillin, ? Streptomycin.		Doran et al. (2015) DMEM/Ham’s F-12, 0.5 mM db-cAMP, 25 mM forskolin, 100 ng/ml NGF, 20 ng/ml GDNF, 10 ng/ml bFGF, 5 mg/ml Y-27623.	
HD10.6 Raymon et al., 1999	Raymon et al. (1999) L-15C, 40 ng/ml FGF-2.	Thellman et al. (2017) and Zhang et al. (2020) DMEM/Ham’s F-12 mix, 1 × GlutaMax, 1 × B-27, 10 ng/ml PGE1, 0.5 ng/ml bFGF, 50 mg/ml G418 solution.	Raymon et al. (1999) L-15C, 10% FBS, 1 mg/ml tetracycline, 2.5% human serum, 5–10 mM forskolin, 25 ng/ml NGF, 25 ng/ml GDNF, 25 ng/ml CNTF, 80 ng/ml heregulin.	Thellman et al. (2017) NeuralQ basal medium, 1 × GlutaMax, GS21 neuronal supplement, 50 ng/ml NGF, 25 ng/ml GDNF, 25 ng/ml CNTF, 25 ng/ml NT-3, 1 mg/ml doxycycline. Zhang et al. (2020) NeuralQ basal medium, 1 × GlutaMax, 1 × B-27, GS21 neuronal supplement, 50 ng/ml NGF, 25 ng/ml GDNF, 25 ng/ml CNTF, 25 ng/ml NT-3, 1 mg/ml doxycycline, 1 μg/ml tetracycline, 25 μM forskolin.
ND-C Wood et al., 1990	Wood et al. (1990) Leibowitz L-15, 10% FCS, 3.3 g/l NaHCO3, ? g/l glucose, 100 U/ml Penicillin. 100 mg/ml Streptomycin. Wu et al. (2008) DMEM, 10% FBS, 50 U/ml Penicillin, 50 mg/ml Streptomycin, 2 mM glutamine.	Rugiero and Wood (2009) DMEM, 10% FBS, 4.5 g/L glucose, ? mM glutamine, 110 mg/L sodium pyruvate, 10,0000 U/ml penicillin-streptomycin.	Wu et al. (2008) DMEM, 0.5% FBS, 100 ng/ml NGF *or* 1 mM db-cAMP. Hackett et al. (2010) Keratinocyte serum-free medium (KSFM) plus bovine pituitary extract and EGF.	

*The table provides an overview over the different components used in maintenance and differentiation media. Question marks are used to indicate absence of information in relation to amounts or concentrations of components. Abbreviations used in the table: Dibutyryl cAMP = db-cAMP; 100 mM hypoxanthine, 1 mM aminopterin, 12 mM thymidine = HAT1 was used to eliminate the parent neuroblastoma cells as the cell line has no HPRT (Platika et al., 1985a); 100 mM hypoxanthine, 0.4 mM aminopterin, 16 mM thymidine = HAT2; Fetal calf serum = FCS; Fetal bovine serum = FBS; 3-Isobutyl-1-methylxanthine = IBMX (phosphodiesterase inhibitor); Dulbecco’s Modified Eagle’s medium = DMEM; 4-(2-hydroxyethyl)-1-piperazineethanesulfonic acid = HEPES; Nerve growth factor = NGF; Glial-derived-neurotrophic-factor = GDNF; Basic fibroblast growth factor = bFGF; Y-27623 = inhibitor of Rho-associated, coiled-coil containing protein kinase (ROCK), Prostaglandin E1 = PGE1.*

Karyological analysis of F-11 cells Platika et al. (1985b) showed that these cells contain concomitant mouse and rat chromosomes and isozymes, and subsequent microarray analysis confirmed the functional presence of DNA from rat and mouse with the demonstration of rat and mouse mRNAs (Zeng et al., 2002; Yin et al., 2016). Out of the initial four clones that possessed neuronal properties including neurite outgrowth, action potentials and labeling with neuron-specific gangliosides, only the differentiated F-11 cell line produced action potentials similar to sensory nociceptive DRG neurons, contained substance P-like immunoreactivity and responded to capsaicin (Platika et al., 1985a, b; Chiesa et al., 1997).

Francel et al. (1987a) further characterized F-11 cells and demonstrated their ability to differentiate in the presence of a medium that contained cAMP increasing molecules (Table 1). Differentiation was accompanied by a reduction in proliferation and the induction of neurite growth. But not all cells differentiated with initially only 25% of cells showing the appropriate morphological characteristics (Boland and Dingledine, 1990a). Increased levels of cAMP induced by application of the cAMP analog db-cAMP or the adenylate cyclase stimulator forskolin cause greater differentiation and the F-11 cells develop long neurites and change expression levels of receptors and channels (Francel et al., 1987a; Boland and Dingledine, 1990a, b; Ghil et al., 2000; Jahnel et al., 2003; Ambrosino et al., 2013; Hashemian et al., 2017). The concentrations of db-cAMP and forskolin necessary to induce complete differentiation of F-11 cells are 0.5 and 10 mM, respectively (Ghil et al., 2000; Gall-Ianotto et al., 2012). Nevertheless, other factors have been shown to successfully differentiate the cells including nerve growth factor, NGF (Martin et al., 2002) and retinoic acid (Naruse et al., 1992). In addition to growth factors, low serum medium containing 1% FBS for about 2 weeks also promoted differentiation (Pastori et al., 2019) (Table 1). In the process of differentiation into a DRG neuron-like phenotype, these cells also change the glycosphingolipid profile that remains different to DRG neurons (Ariga et al., 1995). F-11 cells grow on plastic but for experiments such as Ca^2+^ imaging, cells were grown on poly-L-lysine coated glass coverslips (Hwang et al., 2014).

Undifferentiated F-11 cells are not difficult to culture and passage. However, they start to die after 48–72 h of serum deprivation (0% serum) commonly used in differentiation protocols, and degradation of DNA starts at 24 h, reduction in protein synthesis after 8 h. In the absence of serum, cells still develop neurites which retract after 48 h (Linnik et al., 1993). Interestingly, undifferentiated F-11 cells loose rat and mouse chromosomes during cell division/passages (Cruciani et al., 1994).

RNASeq analysis of undifferentiated F-11 cells confirmed that the cells contained rat and mouse transcript (Table 2). In addition, the analysis described that undifferentiated cells do not represent mature sensory neurons. For example the cells only expressed 9 out of 43 neuronal marker mRNAs (Yin et al., 2016). Another unique characteristic is the high expression of the ganglioside GD3 which seems to be important for the ability of F-11 cells to generate tumors when grafted into nude mice (Zeng et al., 2000a, b, 2002).

**TABLE 2:** Methods utilizing dorsal root ganglion cell lines.

	Transcriptomics	Proteomics and immunohistochemistry	Imaging	Transfection	Electrophysiology and Currents	Others
F-11	Single cell RT-PCR (Jahnel et al., 2003). RT-PCR (Jow et al., 2006). Microarray (Zeng et al., 2002). RNA-Seq (Yin et al., 2016; Martinez et al., 2019; Guo et al., 2020). Cloning of mouse TRPV2 (Bender et al., 2005). Promotor/Luciferase reporter assay (Mahapatra et al., 2006). miRNA detection using graphene-PNA complexes (Oh et al., 2019).	Western Blot (Pollard and volume-sensitive, 1993; Ariga et al., 1995; Wood et al., 1988; Zeng et al., 2000a, 2002; Ross et al., 2001; Gall-Ianotto et al., 2012). Gas chromato-graphic/mass spectrometric analysis (Dasgupta and Banerjee, 1996). Affinity precipitation (Ruan et al., 2008). Immunohistochemistry (Pastori et al., 2019).	^45^Ca^2+^ uptake (Francel et al., 1987a). Fura-2 (Martin et al., 2002). Fluo-4, Poly-D-coated plates (Vetter and Lewis, 2010). Fura-2, glass coverslips (Ambrosino et al., 2013). Fura-2 (Hashemian et al., 2017). FLIPR Calcium-6 (Martinez et al., 2019). Bioluminescence imaging (Hwang et al., 2008). High throughput imaging (Martinez et al., 2019). Live cell imaging and pull down assay (Goswami et al., 2010). NO measurement, DAF-2 imaging (Rimmerman et al., 2008).	Stable transfection (Kim et al., 2003). Transient transfection (Rutter et al., 2005; Goswami et al., 2010; Ambrosino et al., 2013). Adenoviral transfection (Hester et al., 2007). Electroporation (Zhang et al., 2011). Transfection lipofectamine RNAiMAX (Guo et al., 2020).	Whole-cell patch clamp (Boland and Dingledine, 1990b; Ross et al., 2001). Patch clamp workstation (Mollereau et al., 2011). Perforated patch clamp (Ambrosino et al., 2019). Membrane potential measurement (Martinez et al., 2019).	Radioimmunoassay (Platika et al., 1985b). MTT assay (Linnik et al., 1993). HPTLC for glycosphingolipids (Ariga et al., 1995). Rb+ efflux assay (Jow et al., 2006). TrpV1 activation assay (Goswami et al., 2007). cAMP assay and MAPK assay (Fan et al., 2011). Co-culture keratinocytes/F-11 (Gall-Ianotto et al., 2012). EIA assay (Gall-Ianotto et al., 2012). Microfluidic chamber assay (Oh et al., 2019). Dynamic mass redistribution assay (Martinez et al., 2019).
ND7/23	RT-PCR (Tomita et al., 2019). qRT-PCR, miRNAs (Lee et al., 2019; Mukhopadhyay et al., 2019). RNA-Seq (Yin et al., 2016). DNA-affinity pull down assay (Monteiro et al., 2014).	Western Blot. Immunohistochemistry (Ulmann et al., 2007; Wu et al., 2008; Inoue et al., 2012; Binder et al., 2013). Mass spectroscopy (Devaux et al., 2017).	Fura-2 (Ulmann et al., 2007). Fluo-4, Poly-D-coated plates (Vetter and Lewis, 2010; Holmes et al., 2017; Ma et al., 2020).		Whole-cell patch clamp (Zhou et al., 2003, 2019; Morton et al., 2015; Stoetzer et al., 2016). Automated patch clamp platform (Rogers et al., 2016).	Co-culture keratino-cytes/ND7/23 (Ulmann et al., 2007). Time-lapse microscopy (Binder et al., 2013). Luciferase assay (Monteiro et al., 2014). Flow cytometry (Stoetzer et al., 2015). Neurite outgrowth (Mitani et al., 2016; Devaux et al., 2017). Reactive oxygen species (Zhou F. M. et al., 2017; Guo et al., 2018). Biotinylation surface proteins (Zhang et al., 2019). Cell viability (Ulmann et al., 2007; Pires et al., 2017; Akamine et al., 2019; Mukhopadhyay et al., 2019). Cytotoxicity (Nango et al., 2017). TUNEL assay and ROS measurement and Caspase-3 assay (Mukhopadhyay et al., 2019).
ND-C	qRT-PCR (Wu et al., 2008).	Immunocyto-chemistry (Hackett et al., 2010).		Transfection PromoFectin (Solinski et al., 2010). Transfection lipofectamine (Rugiero and Wood, 2009).	Whole-cell patch clamp (Rugiero and Wood, 2009).	
50B11	RT-PCR (Chen et al., 2007; Geng et al., 2009).	Western Blot (Geng et al., 2009; Van Opdenbosch et al., 2012; Zhu et al., 2016; Bestall et al., 2018). Immunohistochemistry (Chen et al., 2007; Pundavela et al., 2014; Hulse et al., 2015; Mohiuddin et al., 2019).	Fura-2, glass coverslips, rat tail collagen (Chen et al., 2007). Fluo-4, Poly-D-coated plates (Vetter and Lewis, 2010). Fluo-4 (Hulse et al., 2015).	Transfection with inactivated virus (Van Opdenbosch et al., 2012). Plasmid transfection using jetPrime reagent (Van Opdenbosch et al., 2012). Knockdown using shRNA (Geng et al., 2009; Bhattacherjee et al., 2017). siRNA (Zhu et al., 2016).	Whole-cell patch clamp (Chen et al., 2007).	^86^Rb uptake (Geng et al., 2009). Thallium uptake in single cells (Geng et al., 2009). Co-culture, transwell chambers (Pundavela et al., 2014). Cell cytotoxicity (Mohiuddin et al., 2019). Superoxide dismutase activity (Mohiuddin et al., 2019). Apoptosis assay (Mohiuddin et al., 2019). Click-iT EdU proli-feration assay (Mohiuddin et al., 2019).
HD 10.6	RT-PCR (Raymon et al., 1999). qRT-PCR (Thellman et al., 2017; Zhang et al., 2020).	Western Blot (Raymon et al., 1999; Thellman et al., 2017). Immunohistochemistry (Thellman et al., 2017; Zhang et al., 2020).			Whole-cell patch clamp (Zhang et al., 2020).	HSV-1 infection (Zhang et al., 2020). Cell viability (Zhang et al., 2020).
MED17.11	RT-PCR (Doran et al., 2015).	Immunohistochemistry (Doran et al., 2015).	Fura-2 Poly-*O*-coated plates (Doran et al., 2015).	Transfection pmax GreenFP (Doran et al., 2015).		

#### Differentiated F-11 Cells Represent a Heterogenous Population of Cells

One question of critical importance is, whether F-11 cells represent a single population of sensory-like neurons. Their morphology certainly suggests more than one subtype of cells in culture, where F-11 cells can be small, flat, round, spindle-shaped or large (Boland and Dingledine, 1990a). Sorting of undifferentiated F-11 cells by flow cytometry showed two main populations of cells, larger, less granular, and smaller cells (Linnik et al., 1993). But what is the effect of differentiation on those subpopulations and the overall function of F-11 cells? The use of various formulations of differentiation media can have profound effects on physiological properties of immortalized cells. Voltage-clamp recordings showed that 25% of cells that were differentiated for 1–3 days showed an increased voltage-activated Ba^2+^ current (Boland and Dingledine, 1990b) and antisera directed against stage-specific embryonic oligosaccharides, SSEA-3 and -4 and B23D8, identified different populations of cells which could be separated by subcloning (Boland and Dingledine, 1990a). Interestingly the staining was independent of the different morphologies of F-11 cells (Boland and Dingledine, 1990a) (Table 2).

#### Expression of Neuronal and Nociceptor-Related Receptors and Molecules

The function of DRG neurons and, in particular, nociceptive neurons is associated with specific subsets of molecules such as receptors, channels and enzymes. Whereas stage-specific embryonic markers suggest heterogeneity, neurofilament 200 (NF200), a marker used to identify large DRG neurones in mice and rats, was present in all differentiated F-11 cells, and no immunoreactivity for markers of satellite and glia cells, GFAP and S-100, could be found in F-11 cultures (Boland and Dingledine, 1990a). Transient receptor potential (Trp) channels are present in nociceptors and Trp channel subtypes are key components of pain signaling (Basbaum and Braz, 2010; Naziroglu and Braidy, 2017). Trp family members are expressed in both naïve nociceptive DRG neurons and F-11 cells. Channel proteins such as TrpV1 or TrpA1 are closely associated with nociceptive signaling. Undifferentiated F-11 cells do not express the Trp channel family members TrpA1 (Yin et al., 2016) and TrpV4 (Goswami et al., 2010) but do express rat and mouse Trpv2 (Jahnel et al., 2003). They also express no or very low levels of TrpV1 mRNA and no transcripts for the nociceptor-associated neuropeptide calcitonin gene-related peptide, CGRP (Yin et al., 2016). The absence of nociceptor-related mRNAs and proteins in F-11 cells changes upon differentiation where mouse but not rat mRNA for TrpV1 is present (Jahnel et al., 2001, 2003; Ambrosino et al., 2013; Yin et al., 2016; Hashemian et al., 2017). In addition to TrpV1, differentiation with retinoic acid and NGF increased also the relative mRNA expression levels for TLR4, cannabinoid and bradykinin receptors (Hashemian et al., 2017) (Tables 1, 3).

**TABLE 3:** Summary of characteristics of immortalized DRG neuron cell lines.

	F-11	ND7/23	ND-C	50B11	MED17.11	HD10.6
Species of origin	Rat DRG E13-14 × mouse neuroblastoma cell line N18TG2.	Rat DRG neonatal × mouse neuroblastoma cell line N18TG2.	Rat DRG neonatal × mouse neuroblastoma cell line N18TG2.	Rat DRG E14.5.	Mouse (H2kbtsA58 Immortomouse) DRG E12.5.	Human DRG embryonic - 1st trimester.
First description (see Table 1)	Platika et al., 1985a	Wood et al., 1990	Wood et al., 1990	Chen et al., 2007	Doran et al., 2015	Raymon et al., 1999
Culture surface and matrix composition	Glass/plastic. Poly-D-lysine. Gelatin	Glass/plastic. Poly-D-lysine.	Glass/plastic, collagen type I. Laminin.	Glass/plastic. No matrix.	Glas/plastic. Poly-L-ornithine.	Glass – Matrigel coated. poly-D-lysine. Plastic - fibronectin-coated.
Differentiation agents used (see Table 1)	db-cAMP, retinoic acid, NGF, BDNF, NT	db-cAMP, retinoic acid, NGF	db-cAMP	Forskolin, NGF, GDNF, estrogen, angiotensin II	Forskolin, NGF, GDNF, bFGF, Y-27623	Forskolin, NGF, GDNF, CNTF, heregulin
RNAseq (see Table 2)	Data available for undifferentiated treated and untreated cells.	Data available for undifferentiated cells.	No data available.	No data available.	No data available.	No data available.
Nociceptor-related molecules and responses (see Table 2)	Present, predominantly in differentiated cells.	Present, predominantly in differentiated cells.	Some molecules and channels present but no functional TrpV1, TrpA1 channels.	Present, predominantly in differentiated cells.	Present, predominantly in differentiated cells.	Present, predominantly in differentiated cells.
Endogenous Ion channel expression	Voltage-gated Ca^2+^ and Na^+^ channels present, differently expressed in undifferentiated and differentiated cells.	Voltage-gated Ca^2+^ and Na^+^ channels present, differently expressed in undifferentiated and differentiated cells.	Voltage-gated Na^+^ channels and mechanosensitive currents.	Indirect evidence for voltage-gated Ca^2+^ channels, no electrophysiology studies conducted.	Indirect evidence for voltage-gated Na^+^ channels, no electrophysiology studies conducted.	Voltage-gated Ca^2+^ and Na^+^ channels present in differentiated cells.
Primary reported use (see Table 2)	Investigation of differentiation and neurite outgrowth.	Transfection and investigation of Voltage-gated Na^+^ channels.	Investigation of mechanosensitivity.	Investigations of toxicity.	Only one descriptive publication.	Investigation of virus transfection.
Experimental considerations	After 5 passages no response to gabapentin, loss of opioid receptors after 10 passages.	Mouse and rat mRNAs and proteins present and expressed.	Has only some characteristics of nociceptors.	Electrophysiological characteristics unknown.	Not available.	Limited description due to low number of publications.

Functional TrpV1 channels have been demonstrated with the inward currents, depolarisation and increase in intracellular Ca^2+^ levels in response to the TrpV1 agonist capsaicin (Platika et al., 1985a; Ambrosino et al., 2019; Pastori et al., 2019) and via blockade of the capsaicin-dependent intracellular Ca^2+^-increase by the antagonist capsazepine (Ambrosino et al., 2013). Early experiments demonstrated the presence of the neuropeptide substance P (SP) but not CGRP via radioimmunoassay in differentiated F-11 cells (Wood et al., 1990). Later it was shown, that F-11 cells that were differentiated in keratinocyte serum-free-medium supplemented with NGF and db-cAMP expressed substance P (SP) and CGRP and released those neuropeptides in response to capsaicin (Gall-Ianotto et al., 2012). Netrin-1 upregulated CGRP mRNA in F-11 cells (Guo et al., 2020). Recent studies also demonstrated that differentiated cells respond to SP with a small inward current (Pastori et al., 2019). Acidic solutions and acetylcholine, but neither capsaicin nor an agonists of the nociceptor-related mas-related gene (Mrg) receptors produced no effects in undifferentiated F-11 cells (Solinski et al., 2010; Yin et al., 2016; Pastori et al., 2019). A high proportion of low-serum differentiated cells responded to glutamate, acetylcholine and acidic solutions (Pastori et al., 2019). In addition to the investigation of endogenous channel proteins, Trp channels and Mrg proteins were also transfected into undifferentiated F-11 cells and investigated (Rutter et al., 2005; Goswami et al., 2007; Solinski et al., 2010).

The presence of molecules in F-11 cells that are characteristic of nociceptors is not restricted to ion channels and peptides. F-11 cells express the endothelial and neuronal isoforms of the nitric oxide synthase (NOS) (Rimmerman et al., 2008) which both have been demonstrated in nociceptive DRG neurons (Henrich et al., 2004; Papadopolou et al., 2004). Activation of NOS with subsequent generation of nitric oxide contributes to distinct pain signaling pathways that lead to central sensitization and also mediate the effects of opioids (Cury et al., 2011).

Opioids and consequently opioid receptors are also innately linked to pain signaling (Francois and Scherrer, 2018). Undifferentiated and differentiated F-11 cells express the opioid receptor subtypes δ and μ but not κ (Fan et al., 1992; Cruciani et al., 1993). Undifferentiated cells showed a ratio for δ and μ surface receptors of 6:1 (Cruciani et al., 1993). Activation of the receptors inhibited adenylate cyclase via *G*_*i/o*_ and reduced intracellular cAMP levels (Francel et al., 1987a; Fan et al., 1992) but the experiments suggested that δ receptor activation might also be coupled to *G*_*s*_-protein signaling (Cruciani et al., 1993). Interestingly, loss of rat and mouse chromosomes in undifferentiated F-11 cells during cell division/passages has been shown to impact on opiate receptor expression. The passage-dependent chromosomal loss caused total absence of δ and μ receptors in F-11 cells after 10 passages (Cruciani et al., 1994).

In addition to opioids, the cannabinoid system is another potential therapeutic target in treatment of pain. Cannabinoid receptors, CB1 and CB2 are expressed in rat DRG neurons (Svizenska et al., 2013; Oliveira-Fusaro et al., 2017) as well as in differentiated and undifferentiated F-11 cells (Ross et al., 2001; Yin et al., 2016; Hashemian et al., 2017). F-11 cells express mouse but not rat CB1 and CB2 mRNA (Hashemian et al., 2017) with the CB1 receptor showing a stronger immunoreactivity in undifferentiated F-11 cells compared to CB2 (Ross et al., 2001).

Bradykinin is a pro-inflammatory mediator that has been shown to modulate pain via activation of two receptor subtypes (BKB1R and BKB2R) present on DRG neurons (Chen et al., 2010; Takemura et al., 2011). Binding sites for bradykinin are present on undifferentiated and differentiated F-11 cells and incubation of undifferentiated cells with bradykinin caused a transient increase in intracellular Ca^2+^ and the *G*_i/o_-dependent production of IP_3_ and DAG (Francel et al., 1987a, b; Francel and Dawson, 1988; Solinski et al., 2010; Vetter and Lewis, 2010; Ambrosino et al., 2019). Even though undifferentiated and differentiated cells possess binding sites for bradykinin, the Ca^2+^ response to bradykinin increased in differentiated F-11 cells (Ambrosino et al., 2019).

The toll-like receptor 4 (TLR4) is a pattern recognition receptor that is expressed by DRG neurons and contributes to inflammatory and chemotherapy-induced pain (Li et al., 2015). F-11 cells express mouse but not rat TLR4 mRNA and protein with increased levels of both in response to differentiation. Isolated and cultured DRG neurons respond to LPS-mediated TLR4 activation with massive increases in interferon β, TNF-α or Il-6 expression but LPS had no effect in differentiated and undifferentiated F-11 cells (Hashemian et al., 2017).

Lipids were also investigated in F-11 cells. Depolarisation of F-11 cells also increased production of the lipid *N*-palmitoyl glycine (PalGly) in undifferentiated cells where the lipid also caused Ca^2+^ influx (Rimmerman et al., 2008).

#### Ion Channels

F-11 cells express a large variety of ion channels. RNA analysis demonstrated the presence of 223 mouse and 201 rat ion channel genes in undifferentiated F-11 cells (Yin et al., 2016) (Table 2). Voltage-gated calcium channels (VGCCs) are key components in pain signaling. Activation of nociceptors includes opening of Ca^2+^-channels, and drugs that reduce pain such as opioids often block Ca^2+^-channels. VGCCs can be divided into high-voltage-activated (HVA) and low-voltage-activated (LVA) channels. HVA channels are composed of different subunits, α, β, and γ, whose composition determines types of Ca^2+^ currents (L-type, N-type, P/Q-type, and R-type). The α-subunits are often related to a specific current type, e.g., the α1-subunit Ca_V_2.1 is related to the P/Q-type current. LVA channels represent the T-type current (Altier and Zamponi, 2008). Neither undifferentiated nor cells differentiated under low-serum conditions expressed LVA channels (Pastori et al., 2019). Undifferentiated F-11 cells express mRNAs for all calcium channel a-subunit (Ca_V_) isoforms with Ca_V_3.2 expressed at the highest level (Yin et al., 2016). Functionally, early and recent studies showed that F-11 cells possess endogenous L-type Ca^2+^-channels but these were 5-10 times less dense compared to cultured rat DRG neurons (Boland and Dingledine, 1990b; Pastori et al., 2019). Early studies also suggested that Ca^2+^-channels expression was higher in differentiated compared to undifferentiated cells (Francel et al., 1987a). In addition to L-type channels, the cells also possess N-type like Ca^2+^-channels. Functional consequences of depolarisation and subsequent Ca^2+^-channel activation have been demonstrated by the release of SP-like immunoreactivity from differentiated and undifferentiated F-11 cells (Boland and Dingledine, 1990b). Depolarisation of F-11 cells with KCl caused subsequent activation of VGCCs and increase in intracellular Ca^2+^-levels (Martin et al., 2002). This response was stronger in differentiated compared to undifferentiated cells and the robust Ca^2+^-response of differentiated F-11 cells to depolarisation was used for screening of analgesics and investigations into the function of the anticonvulsant and inhibitor of α2δ-subunits containing VGCC, gabapentin (Martin et al., 2002; Martinez et al., 2019). Gabapentin (applied 1–2 min before depolarisation) caused a small dose-dependent reduction of the depolarisation-induced Ca^2+^ influx into F-11 cells (Martin et al., 2002). Interestingly, the response was dependent on passage number as passage numbers > 5 did not respond to gabapentin. Non-responding cells showed marked differences in the relative mRNA expression levels of β_2_ and α2δ2- VGCC subunits with a higher expression of β_2_ and a lower expression of mRNA for α2δ2 in gabapentin-responsive cells.

With the exception of VGCCs, very few studies investigated changes in ion channel expression levels in F-11 cells. Early studies from Boland and Dingledine that were confirmed by Pastori et al. (2019) demonstrated that all Na+ currents in differentiated and undifferentiated F-11 cells could be blocked by tetrodotoxin (TTX), indicating that the majority of voltage-gated sodium channels (VGSCs) belongs to the class of TTX-sensitive channels (Boland and Dingledine, 1990b; Clement et al., 2007; Wang et al., 2017; Pastori et al., 2019). In addition to TTX, a toxin of the spider *Grammostola rosea* was also able to block Na^+^ currents in F-11 cells (Clement et al., 2007). Recently, F-11 cells were used to investigate voltage-gated potassium channels of the Kv7 subgroup. The channels are contributing to the I_KM_ current in nociceptive DRG neurons and are part of TrpV1 signaling. All known Kv7 subunits of the channels were expressed in F-11 cells and a current with similarities to I_KM_ was identified (Ambrosino et al., 2019). Additionally, both undifferentiated and differentiated cells also express the ERG potassium current with increased current density observed in differentiated cells (Faravelli et al., 1996; Pastori et al., 2019).

#### Differentiation and Neurite Outgrowth

F-11 cells represent undifferentiated sensory neurons but respond to stimuli such as growth factors with changes in cell morphology and neurite outgrowth. Therefore, the cell line was utilized for research into the process of sensory neuron differentiation. Neurite outgrowth and neuronal marker expression in undifferentiated F-11 cells in response to different agents were used as read-outs. When exposed to differentiation medium, neurite formation started after 3 h. Initial studies indicated that after 72 h no new neurite formation could be observed (Francel et al., 1987a) but later it was demonstrated that neurite extension and differentiation could be still demonstrated at day 6 (Gall-Ianotto et al., 2012). Differentiation of F-11 cells in keratinocyte serum-free-medium supplemented with NGF and db-cAMP induced neurite outgrowth that could not be increased by addition of B27 or N2 supplement or addition of BDNF and/or NT-3 (Gall-Ianotto et al., 2012). Recently, long term exposure (12–14 days) to low-serum (1% FCS) without addition of growth factors achieved differentiation in 50% of cells. Compared to undifferentiated cells a significantly higher proportion of low-serum differentiated cells generated action potentials in response to stimulation (Pastori et al., 2019) (Table 1).

Novel approaches such as the use of optical imaging reporters to monitor neuronal differentiation processes were utilized to investigate the roles of the transcription factor neurogenin 1 (Ngn1) (Oh et al., 2013). Ngn1 induced neurite outgrowth in undifferentiated F-11 cells and increased the relative mRNA expression levels of neuronal markers Tuj-1, NeuroD, MAP2 and neurofilament-M but not of NeuN (Oh et al., 2013). Further investigations showed a role for the microRNAs miR-124 and miR193a in this process. Increase in miR-124 levels increased neurite outgrowth as well as immunoreactivities for Tuj1 and neurofilament (Jang et al., 2016) whereas miR193a was able to increase the efficiency of Ngn1-mediated differentiation by down-regulating proliferation associated mRNAs. Interestingly, studies investigating Ngn1 and miRNAs also demonstrated the intercellular transport and uptake of miR-193a via exosomes (Oh et al., 2017; Oh et al., 2019).

In addition to the investigation of basic mechanisms underlying neuronal differentiation, drugs and peptides were also investigated to their potential to induce neurite outgrowth. The antidepressant fluoxetine hydrochloride and activation of transfected TrpV4 channels reduced neurite outgrowth and induced growth cone retraction in forskolin-differentiated F-11 cells (Goswami et al., 2010; Xu et al., 2010) whereas the peptide analog PACAP38 caused an 170-fold increase in neurite outgrowth compared to forskolin or db-cAMP (McIlvain et al., 2006) (Table 2). A factor produced by macrophages, netrin-1, also increased length and number of neurites in F-11 cells (Guo et al., 2020).

In summary, F-11 cells are widely used in neuroscience and research has utilized a magnitude of different methods to describe the presence and function of neuron-associated mRNAs, miRNAs and proteins in undifferentiated as well as the differentiated F-11cells. F-11 cells express many molecules that are characteristic for sensory neurons and specific for nociceptors, with expression profiles that are highly dependent on differentiation state. A comprehensive analysis of transcripts has only been done for undifferentiated cells. Similarly, at the protein level a lack of comprehensive proteomics in undifferentiated and differentiated cells does not allow conclusions to be drawn with respect to the translational profile of F-11 cells. The cells are widely used for analysis of signaling and neurite outgrowth but the documented presence of mouse and rat mRNAs and proteins in the cell line and the loss of chromosomes when passaged over a longer period are potential limitations should be considered.

### ND7/23 Cells

ND7/23 cells were generated by fusion of cultured neonatal rat DRG neurons with N18TG2 mouse neuroblastoma cells in serum-free L-15 medium containing 50% PEG_1500_ (Wood et al., 1990). Undifferentiated cells are round and have no neurites (Wood et al., 1990; Zhang et al., 2017) but maintain neuronal identity and can be stained with neuronal markers such as PGP9.5, MAP1 and Smi 312 (Ulmann et al., 2007). Differentiation was initially induced by db-cAMP and NGF in L15 medium supplied with 0.5% FCS (Tables 1, 3) and treatment induced neurite outgrowth within 24 h (Wood et al., 1990). The differentiation response to the increase in cAMP was recently confirmed but was different depending on the differentiation medium (Martinez et al., 2019) (Table 1). Differentiated cells were usually used for between 3 and 14 days (Dunn et al., 1991; Zhang et al., 2017; Amaye et al., 2018; Zhang et al., 2019). Differentiated and undifferentiated ND7/23 cells grow on glass and plastic and on uncoated and poly-D-lysine coated surfaces. High glucose concentrations (≥60 mM) lead to DNA-damage and apoptosis of ND7/23 cells. This could be prevented by use of the caspase inhibitor Ac-LETD-CHO and the a-ketoglutarate analog DMOG (Mukhopadhyay et al., 2019). Another hyperglycemia-related molecule, the hydroxyaldehyde glycolaldehyde exhibited toxicity and also induced c-jun terminal kinase and p-38 MAPK-associated cell death in undifferentiated ND7/23 cells (Akamine et al., 2019).

#### Expression of Neuronal and Nociceptor-Related Receptors and Molecules

Differentiated ND7/23 cells, similar to F-11 cells, seem to represent a heterogenous population of cells as subpopulations of cells respond differently to the pro-inflammatory mediator and activator of sensory neurons, bradykinin (Wood et al., 1990; Dunn et al., 1991; Ulmann et al., 2007; Ma et al., 2020). Bradykinin exposure depolarised these cells and induced an inward current as well as ^45^Ca^2+^ and Rb^2+^ efflux in differentiated cells (Wood et al., 1990; Dunn et al., 1991; Ulmann et al., 2007) (Table 2). Interestingly, this response was the opposite to F-11 cells, which responded with an outward current to bradykinin (Wood et al., 1990). Recently it has been shown that differentiated ND7/23 cells showed a stronger increase in intracellular calcium in response to bradykinin when exposed to conditioned medium collected from hypoxia-stressed intervertebral disks (Ma et al., 2020).

ND7/23 cells were utilized as a model to test and to explore adenovirus vector-mediated and chitosan-based gene delivery in nerve cell. The cells showed robust expression of β-galactosidase in response to adenovirus vector transfection (Sivasubramaniam et al., 1997) as well as internalization of nanoparticles (Oliveira et al., 2010a, b) (Table 2). RNAs and proteins were investigated by next generation sequencing (RNA-Seq) and mass spectrometry (MS) and tandem MS, but those studies only investigated undifferentiated ND7/23 cells (Yin et al., 2016; Devaux et al., 2017).

Neuropeptides such as calcitonin-gene related peptide (CGRP) or substance P (SP) are characteristic molecules in subpopulations of nociceptors (Basbaum and Braz, 2010; Haberberger et al., 2019). Radioimmunoassays and immunohistochemistry showed that undifferentiated and differentiated ND7/23 cells contain SP but not CGRP (Wood et al., 1990; Ulmann et al., 2007) (Table 2). Another molecule that is used to label nociceptive neuron subpopulations is the isolectin B4 (IB4) which in mice labels NGF-independent, non-peptidergic nociceptors. Incubation with IB4 labeled undifferentiated ND7/23 cells (Ulmann et al., 2007; Inoue et al., 2012). ND7/23 cells did not respond to GABA, another peptide that has been shown to interact with nociceptors (Suburo et al., 1992).

Regarding nociceptive Trp channel expression, undifferentiated ND7/23 cells showed no Ca^2+^ response to agonists of TrpV1 and TrpA1 channels, a finding that was supported by very low or absent mRNA expression levels for the Trp proteins (Ulmann et al., 2007; Yin et al., 2016). Nevertheless the cells were able to take up the TrpV1 activator anandamide (Thors and Fowler, 2006). Studies using RT-PCR showed that undifferentiated and differentiated cells express TrpC mRNAs 3–7 with increased Trpc4 levels in differentiated cells (Wu et al., 2008), whereas RNA-Seq analysis could not detect TrpC4 and 6 in undifferentiated cells (Yin et al., 2016). In addition to TrpV1, the P_2_X_3_ receptor for ATP is characteristic for subtypes of nociceptors. Undifferentiated ND7/23 cells showed no functional response to the P_2_X receptor agonist αβ-meATP, whereas activation of P_2_Y receptors caused release of Ca^2+^ from intracellular stores (Ulmann et al., 2007) (Table 2). The responses of differentiated cells have not been investigated so far.

Formaldehyde directly activates nociceptors and is used in animal models of acute pain. Undifferentiated ND7/23 cells were used as a model of nociceptors and responded to formaldehyde with an dose-dependent increase in intracellular Ca^2+^ (Fischer et al., 2015).

Acid-sensing ion channels (ASICs) are present on nociceptive DRG neurons and are involved in nociceptive signaling in for example cancer related pain (Lozano-Ondoua et al., 2013). Indirect evidence for the presence of ASICs in differentiated ND7/23 cells was provided by the activation of currents using solutions with a low pH (Moore et al., 2005; Ma et al., 2020).

Serotonin, 5-hydroxytryptamine (5-HT), activates nociceptive DRG neurons and induces pain or itch via interaction with 5-HT receptors (Hachisuka et al., 2010; Lin et al., 2011). 5-HT_3_A receptors are involved in pain signaling (Costall and Naylor, 2004) and belong to the class of ligand-gated ion channels that consist of 5-HT_3_A homopentamers. Undifferentiated ND7/23 cells express endogenous 5-HT_3_A receptors (Morton et al., 2015). Stimulation of the receptors increased the 5-HT_3_A-mediated current density in ND7/23 cells (Morton et al., 2015).

Sensory DRG neurons are important components of itch signaling (Akiyama and Carstens, 2013). Undifferentiated ND7/23 cells respond, similar to sensory DRG neuron subpopulations, to the itch-inducing molecules chloroquine and compound 48/80 (Zhou F. M. et al., 2017) and this chloroquine- and compound 48/80-dependent increase in intracellular Ca^2+^ was decreased by the major green tea polyphenol EGCG (Guo et al., 2018).

#### Ion Channels

RNA analysis demonstrated the presence of 214 mouse and 189 rat ion channel genes in undifferentiated ND7/23 cells (Yin et al., 2016) including calcium and sodium channels. Based on the activation threshold, VGCCs can be divided into HVA and LVA channels. Undifferentiated ND7/23 cells only expressed a transient LVA current whereas 40–50% of differentiated cells showed LVA and HVA currents (Kobrinsky et al., 1994; Zhang et al., 2017). Differentiation also reduced the activation voltage necessary to induce maximal VGCC opening (Zhang et al., 2017).

ND7/23 cells showed a prominent T-type Ca^2+^-current and expressed the related Ca_V_3.2 channel proteins (Table 3). The T-type current increased upon differentiation (Mitani et al., 2016; Zhang et al., 2017; Amaye et al., 2018) but interestingly independent of the presence of NGF in the differentiation medium (Zhang et al., 2017). The anticancer drug Bortezomib increased Ca_V_3.2 channel proteins and T-type currents in ND7/23 cells (Tomita et al., 2019).

Voltage-gated sodium channels (VGSCs) are an additional key component of neuronal excitability. They consist of nine different pore forming α-subunits in combination with four auxiliary β-subunits. Based on the nine α-subunits nine VGSCs, Na_V_1.1-Na_V_1.9, have been described (Altier and Zamponi, 2008; Wang et al., 2017). Many studies used ND7/23 cells for transfection and expression of sodium channels. However, RT-PCR and functional studies showed that undifferentiated ND7/23 cells already express endogenous VGSCs with the majority of channels being TTX-sensitive. Na_V_1.7 and Na_V_ 1.6 are major subtypes with Na_V_1.2, Na_V_ 1.3 and Na_V_ 1.9 channels contributing only little to activity. Na_V_1.7, Na_V_ 1.6 and Na_V_ 1.9 represent the mouse channel proteins. The accessory subunits β1 and β3 are expressed together with pore-forming α-subunits (John et al., 2004; Leffler et al., 2010; Kennedy et al., 2013; Rogers et al., 2016; Lee et al., 2019). Interestingly, VGSC were not investigated in differentiated cells. Endogenously expressed Na_V_1.7 and other VGSC were targeted in undifferentiated ND7/23 cells to investigate the mechanisms underlying actions of pain modulating molecules such as local anesthetics, μ-opioid receptor agonists or the toxin bufalin (Leffler et al., 2012; Stoetzer et al., 2017; Tao et al., 2018) but also the antidepressant duloxetine and methadone which blocked endogenous sodium currents (Stoetzer et al., 2015, 2016). The impact of toxins isolated from the venom of the Asian scorpion *Buthus martensi* Karsch on endogenously expressed VGSC in undifferentiated ND7/23 cells was investigated to explain the pain-related effects of the venoms (Zhu et al., 2009, 2015; Feng et al., 2015).

A large number of studies have used ND7/23 cells for the stable and transient transfection of proteins, in particular for investigations of different isoforms and mutations of sodium channels (for example Zhou et al., 2003, 2018, 2019; Dong et al., 2007; Leffler et al., 2010; O’Brien et al., 2012; Vanoye et al., 2013; de Kovel et al., 2014; Savio-Galimberti et al., 2014; Morton et al., 2015; Stoetzer et al., 2015; Wagnon et al., 2017; Wu et al., 2017; Zhou F. M. et al., 2017; Zhou X. et al., 2017; Dash et al., 2018; Li et al., 2018). Most studies transfected cells and electrophysiology was performed after 24–48 h. The reason for the popularity of ND7/23 cells in VGSC research is the endogenous expression of channel-associated proteins in undifferentiated cells which enables and facilitates the incorporation of functional channel proteins into membranes and establishes electrophysiological characteristics that mimic those of naïve channels (Rogers et al., 2016). This review only describes the endogenous expression of ion channels.

#### Differentiation and Neurite Outgrowth

Compared to F-11 cells few publications have utilized the ND7/23 cell line for investigating differentiation and neurite outgrowth. However, it has been shown that undifferentiated cells give rise to short neurites after 48 h whereas differentiation induces outgrowth of long unbranched neurites (Wu et al., 2008). Extracellular Ca^2+^ is necessary for axon growth but co-culture experiments with keratinocytes rescued axonal growth in the presence of low extracellular Ca^2+^ levels (Ulmann et al., 2007). Notably, Mitani et al. (2016) showed that the prostaglandin PGE_2_ and the second messenger cAMP cause neurite outgrowth via the PGE_2_/EP_4_/cAMP signaling under participation of protein kinase A and Ca_V_3.1 channels. Inhibition of T-type Ca^2+^-channels caused a reduction in the number and length of dendritic processes induced by differentiation (Zhang et al., 2017). Expression of the TrpC4 protein was shown to be associated to growth of processes in differentiated ND7/23 cells as shRNA against TrpC4 blocked db-cAMP-induced neurite outgrowth (Wu et al., 2008). The impact of extracellular matrix proteins such as electrospun fibers on neurite outgrowth from differentiated ND7/23 cells was shown with the positive effect of polylactic-glycolic acid fibers (Binder et al., 2013). The involvement of the NGF receptor TrkA in neurite outgrowth was demonstrated in co-culture experiments with keratinocytes. Co-culturing induced neurite outgrowth which was blocked by the TrkA antagonist K252a (Ulmann et al., 2009). Recently it was demonstrated that the neuronal guidance molecule netrin-1 significantly increased neurite outgrowth in undifferentiated F-11 cells (Guo et al., 2020).

The role RhoA signaling and RhoA inhibitors in axon growth were investigated in differentiated (Pires et al., 2017) and undifferentiated NF7/23 cells (Devaux et al., 2017). RhoA inhibitors improved neurite outgrowth in ND7/23 cells that were conditioned with medium from injured spinal cord (Devaux et al., 2017). The neurite outgrowth promoting drug zonisamide induced the phosphorylation of AKT and Erk1/2 (Takaku and Sango, 2020).

In summary, the endogenous presence of accessory subunits that are necessary to express functional ion channels makes ND7/23 cells the working horse of VGSC research. Despite their presence, most papers do not refer to endogenous VGSCs and changes in expression profiles and function of ion channels that occur after differentiation and maturation of ND7/23 cells have not been investigated so far. There are obvious differences in cell function between undifferentiated and differentiated cells. It is not clear if the heterogenous population of undifferentiated ND7/23 cells differentiates into multiple phenotypes or acquires a more homogenous type of sensory neuron-like cell. Interestingly, even though ND7/23 express a variety of nociceptor-related molecules and are used as models of nociceptors, characteristic channel proteins such as TrpV1 or TrpA1 are absent in undifferentiated cells and have not been investigated in differentiated cells.

### ND-C Cells

In addition to ND7/23 cells, ND-C cells were also generated by fusion of cultured neonatal rat DRG neurons with N18TG2 mouse neuroblastoma cells in serum-free L-15 medium containing 50% PEG_1500_ (Wood et al., 1990). Cells were differentiated using db-cAMP and NGF (Wu et al., 2008), and also differentiated using keratinocyte serum free medium (KSFM) (Hackett et al., 2010) (Tables 1, 3). Co-culture experiments demonstrated the ability of ND-C cells to form contacts with other cells such as C2C12 myocytes and to induce epithelial cell proliferation (Hackett et al., 2010).

#### Expression of Neuronal and Nociceptor-Related Receptors and Molecules

Early studies showed that opioids or d-receptor agonists increased intracellular Ca^2+^ levels in ND-C cells via activation of dihydroperidine-sensitive channels (Tang et al., 1994). In later studies, the cells were analyzed for the presence of TrpC mRNAs and used for transfection of TrpC4. Cells were differentiated in the presence of db-cAMP or NGF and showed increased relative mRNA expression for TrpC4, 5, 6, and 7. TrpC1 was present in undifferentiated cells and did not change and TrpC3 could not be detected (Wu et al., 2008). Differentiated ND-C cells responded to acid but not to the TrpV1 agonist capsaicin which indicates the presence of ASIC-like channels but not TrpV1 (Rugiero and Wood, 2009). In addition to capsaicin and 2-ABP, the cells were also insensitive to the TrpM8 agonist menthol, the TrpA1 activators cinnamaldehyde and mustard oil, and the TrpV4 agonist 4a-PPD. This suggests that the cells have no nociceptive phenotype. Interestingly, ND-C cells were shown to be mechano-sensitive (Rugiero and Wood, 2009).

#### Ion Channels

Electrophysiology using ND-C cells showed that the cells possess VGSCs which were TTX-sensitive (Rugiero and Wood, 2009) and identified chloride and potassium channels responsible for the hyperpolarisation-gated inward current (Hackett et al., 2010).

#### Differentiation and Neurite Outgrowth

Cells were differentiated using db-cAMP and NGF (Wu et al., 2008) and a differentiation using KSFM medium supplemented with bovine pituitary extract and EGF differentiated 10% of cells (Hackett et al., 2010) (Table 1). When KSFM medium was used, db-cAMP, NGF and dexamethasone had no significant additional effect on differentiation (30–50% of cells) but increased neurite length (Table 1). Differentiation on collagen-based matrices induced the growth of processes longer than 200 mM in 1/5th of all cells (Hackett et al., 2010).

In summary, ND-C is a sensory DRG neuron cell line that shares functional similarities with mechanosensitive rather than nociceptive neurons (Table 3). This represents a unique opportunity to utilize these cells for investigations of mechanosensitivity.

### 50B11 Cells

The 50B11 cell line was generated by electroporation and transfection of the SV40 large antigen and subsequent transfection with the human telomerase reverse transcriptase, hTERT, into primary cultured rat embryonal (E14.5) DRG neurons (Chen et al., 2007). Cells were grown in Neurobasal medium containing 10% FBS, 0.5 mM glutamine, 0.2% glucose and B-27 supplement (Tables 1, 3). 50B11 cells grow on uncoated plastic dishes and have a replication rate of about 36 h (Chen et al., 2007). The initial description used a combination of forskolin, NGF and GDNF to start differentiation (Chen et al., 2007). Differentiation induced neurite outgrowth and changed cell morphology. The changes could be induced by forskolin alone and was most stable 20–36 h post forskolin (Bhattacherjee et al., 2014).

#### Expression of Neuronal and Nociceptor-Related Receptors and Molecules

Differentiated 50B11 cells could be labelled with pan-neuronal markers such as III-tubulin (Chen et al., 2007; Bhattacherjee et al., 2014) and PGP9.5 (Bhattacherjee et al., 2014; Pundavela et al., 2014). In addition, different receptors that are characteristic for nociceptors have been shown to be expressed in 50B11 cells. TrpV1 and TrpA1 receptors are functional in differentiated 50B11 cells as TrpV1 agonist capsaicin and the TrpA1 receptor agonist allyl isothiocyanate, AITC, increased levels of intracellular Ca^2+^ (Chen et al., 2007; Vetter and Lewis, 2010) (Table 2). Undifferentiated cells could be activated by ATP but failed to respond to other agents such as acetylcholine and bradykinin (Vetter and Lewis, 2010).

Differentiated 50B11 cells express in addition to TrpV1 other molecules typical for nociceptors such as peripherin, a molecule characteristic for small C-fiber type sensory neurons. Receptor proteins for the nociceptor-related growth factors NGF and GDNF such as trkA, p75NTR, c-Ret, GFRa1, and GFRa2 were detected in differentiated cells using RT-PCR and immunohistochemistry (Chen et al., 2007; Bhattacherjee et al., 2014). Consistent with this, the cells respond to NGF and GDNF (Chen et al., 2007; Bhattacherjee et al., 2014) (see below).

A key transporter in sensory nociceptive neurons is the Na^+^-K^+^-2Cl^–^ cotransporter (NKCC1). This transporter together with other cation-chloride cotransporters and potentially regulatory kinases was detected in undifferentiated 50B11 cells via RT-PCR (Geng et al., 2009). Proteins for the kinases SPAK and OSR1 were also present in 50B11 cells and modulated NKCC1 activity (Geng et al., 2009).

#### Ion Channels

Ion channels are certainly present in 50B11 cells. This is supported by the use of calcium imaging to show activity of cells, but individual channels have so far not been investigated and described using patch-clamp or immunohistochemistry (Table 2). Interestingly, action potentials (AP) could be initiated only in differentiated cells. The only publication that described the ability to generate APs was the initial description of 50B11 cells (Chen et al., 2007).

#### Differentiation and Neurite Outgrowth

50B11 cells are easy to grow. They grow on uncoated plastic or glass as well as on poly-L-lysin coated surfaces (Chen et al., 2007; Bhattacherjee et al., 2017). 50B11 cells were tested for their ability to grow neurites along artificial extracellular matrices. It was demonstrated that these cells grow and differentiate on 300 nm gelatin and chitosan fibers (Gnavi et al., 2015).

Neurite outgrowth and inhibition of proliferation (as markers of differentiation) could be induced by addition of forskolin usually in a concentration of 75 mM (Van Opdenbosch et al., 2012; Bhattacherjee et al., 2014; Gnavi et al., 2015; Hulse et al., 2015; Bestall et al., 2018) although concentrations of 5, 10, and 50 mM forskolin have been used as well (Chen et al., 2007; Pundavela et al., 2014; Mohiuddin et al., 2019). Forskolin is sufficient to induce neurite outgrowth and establishes and maintains a neuronal phenotype between 20 and 36 h after treatment (Chen et al., 2007; Geng et al., 2009; Bhattacherjee et al., 2014; Hulse et al., 2015).

However, compounding changes to the morphology of 50B11 cells can also be induced by the addition of a variety of growth-inducing factors such as NGF, GDNF, as well as pro-NGF, estrogen and angiotensin II 17 h after forskolin treatment additionally stimulated neurite outgrowth (Bhattacherjee et al., 2014; Pundavela et al., 2014). In addition to differentiating agents, neurite outgrowth in 50B11 cells could also be regulated by knockdown of proteins. Knockdown of the epigenetic controller methyl-CpG binding protein MeCP2 increased neurite outgrowth in differentiated 50B11 cells (Bhattacherjee et al., 2017).

Neurite outgrowth seemed also to be affected by the concentration of glucose in the differentiation medium. The working concentration for glucose in the maintenance and differentiation medium is already high. However, lower as well as higher glucose concentrations inhibited neurite outgrowth (Hulse et al., 2015; Bestall et al., 2018). Differentiation and neurite outgrowth was accompanied by changed mRNA expression levels of receptors for estrogen and angiotensin II (Bhattacherjee et al., 2014).

50B11 cells were also used to investigate the effect of cancer drugs. Studies that investigated the effects of the platinum-based anti-cancer chemotherapeutic cisplatin on 50B11 cells used differentiated (Vencappa et al., 2015) and undifferentiated (Zhu et al., 2016) cells. Cisplatin reduced the protein levels of ATF-3 and the growth factor VEGF-A_165_ and reduced neurite length. Cisplatin also increased levels of caspase 3, ataxin-2 and the spliceosome protein SF3B2.

In summary, 50B11 cells, particularly in the differentiated state, express a variety of sensory neuron and nociceptor-related molecules and seem to represent a suitable model for studies on neurite outgrowth (Table 3). Genomic sequencing and proteomic characterizing have not been conducted using the cells so far and in addition, the presence and function of ion channels in differentiated and undifferentiated cells has not been described.

### MED17.11 Cells

The mouse embryonic DRG cell line, MED17.11, is a conditionally immortalized mouse cell line. It was generated by using DRG neurons from the H2kbtsA58 Immortomouse, that were isolated from E12.5 DRG (Doran et al., 2015). The cells started to change morphology to bipolar cells after few hours in differentiation medium which contained a cocktail of growth factors (bFGF, NGF, and GDNF), forskolin, db-cAMP, and the ROCK inhibitor Y-27632 (Tables 1, 3). Differentiation was dependent on cell density with 5,000–15,000 cells/cm^2^ produced the best differentiation.

#### Expression of Neuronal and Nociceptor-Related Receptors and Molecules

Undifferentiated cells could be stained for the pan-neuronal markers Tuj1 and FOX3 as well as for the marker of sensory nociceptive neurons, Isl1. The cells were also immunoreactive for the sensory neuron marker advillin. Differentiation induced the mRNA expression of sensory neurons and nociceptor-specific transcription factors FOXS1 and RUNX1. mRNAs for the GDNF receptor cRET, the neuropeptide CGRP and the NGF receptors TrkA and TrkC could be detected (Table 2). The Trp channel mRNAs for TrpV1 and TrpV2 were present in differentiated cells whereas TrpA1 mRNA could not be amplified and the TrpA1 agonist cinnamaldehyde had no effect. There was no evidence for functional Trpm8 receptors. Characteristic mRNAs for mechanosensitive receptors, Piezo 1 and 2, were also detected. MED17.11 cells could be labeled with IB4 and contained not only mRNA but also showed immunoreactivity for TrkA and TrkC. More than half of differentiated cells responded to bradykinin, but only about 1/3 to histamine and noradrenaline indicating a functionally heterogenous population of cells (Doran et al., 2015).

#### Ion Channels

The mRNAs for VGSCs Na_V_1.7 and Na_V_1.9 but not Na_V_1.8 were detected only in differentiated cells. In addition, immunoreactivity for Na_V_1.3 was present. The presence of TTX-sensitive VGSCs was supported by activation of the channels with veratridine which caused an increase in Ca^2+^. The presence of VGCCs is likely, as indicated by the increase in intracellular Ca^2+^ in response to depolarisation with KCl.

The single publication using MED17.11 cells did not conduct experiments related to neurite outgrowth.

### HD10.6 Cells

In contrast to other sensory neuron-derived cell lines, HD10.6 cells are immortalized human DRG cells. The cell line was first described by Raymon et al. (1999). Cultures of embryonic human DRG were immortalized using LINX *v-*myc as a retroviral vector. Undifferentiated cells divided about every 24 h but were not able to generate action potentials. Using tetracycline (doxycycline) to stop ongoing *v*-myc transcription and using L15-C or Ultraculture medium containing a cocktail of growth factors (Tables 1, 3), cells were differentiated and differentiation was complete after 3–4 days (Raymon et al., 1999). Cells grow on fibronectin or on poly-D-lysine and Matrigel coated coverslips (Zhang et al., 2020). Twenty years after the initial description, a second study used the cell line to investigate herpes simplex virus infection (Thellman et al., 2017). Short tandem repeat profiling defined the cell line as human, male. Modifications of the differentiation medium (Table 1) had no negative effect on nociceptive neuron marker expression (Thellman et al., 2017).

#### Expression of Neuronal and Nociceptor-Related Receptors and Molecules

The HD10.6 clone showed characteristics of sensory neurons such as b-III tubulin immunoreactivity and immunoreactivities for neurofilament 160 (NF160) as well as peripherin in large populations of differentiated cells. Subpopulation of differentiated cells also contained nociceptor-related molecules such as the neuropeptide substance P, the NGF receptor proteins TrkA and p75. HD10.6 cells expressed the transcription factors DRG11, a factor characteristic for post-migratory sensory neurons. Half of the differentiated cells expressed another DRG-specific transcription factor, Islet-1 (Raymon et al., 1999). In contrast to the initial description, where TrkB, TrkC, and RET could not be detected, strong expression of TrkA, TrkB, TrkC, and RET receptors was described in HD10.6 cells 7 and 14 days post-differentiation (Thellman et al., 2017).

#### Ion Channels

Differentiated HD10.6 cells expressed TTX-sensitive voltage-gated sodium channels, HVA Ca^2+^-channels, αβ-methylene-ATP sensitive purinergic P_2_X receptors and are sensitive to capsaicin. Recent studies confirmed the presence of TTX-sensitive channels with elimination of about 90% of sodium currents using 250 nM TTX and the detection of Nav1.7 mRNA and protein in differentiated HD10.6 cells (Zhang et al., 2020). Capsaicin responses could be blocked by capsazepine indicating the presence of TrpV1 channels (Raymon et al., 1999; Thellman et al., 2017) (Table 2).

#### Differentiation and Neurite Outgrowth

HD10.6 cells were able to differentiate in the presence of different growth factors (Table 1). Differentiation was induced in the presence of human serum, FBS, retinoic acid, forskolin, GDNF, CNTF, NGF, and heregulin b1. Replacement of forskolin with retinoic acid had the same effect (Raymon et al., 1999). The recent study used the growth factors NGF, GDNF, CNTF, and neurotrophin-3 for differentiation (Thellman et al., 2017). Differentiation included neurite outgrowth which was not further investigated.

In summary, the initial description clearly demonstrated an immortalized DRG neuron cell line that has a variety of nociceptor characteristics (Table 3). Almost 20 years later a second study used RT-PCR, immunohistochemistry and Ca^2+^-imaging to further describe HD10.6 cells and use the cells as a model of sensory neurons in the context of herpes simplex virus infection (Thellman et al., 2017).

## Discussion

The development of immortalized DRG-derived cell lines has added numerous useful *in vitro* tools targeted at studies ranging from analysis of ion channel function to the description of intercellular RNA transfer via exosomes (Table 2). While individual cell lines have in each instance been developed by individual investigators seeking desirable characteristics that lead to their use for a specific set of methodologies, there is significant scope for increasing the utilization of DRG-derived cell lines in high-throughput screening tests investigating nociceptor targeted drug screens as well as basic nociceptor biology (Table 2). In most instances this would require additional characterisation of each cell line to better understand their suitability for different experimental approaches. ND7/23 cells for example have been mostly used for investigations of voltage gated sodium channel function, whereas F-11 cells are frequently used for investigations that involve neurite outgrowth and Ca^2+^-imaging (Table 2). The cell lines usually express molecules characteristic for sensory neurons and nociceptors such as neuropeptides CGRP or SP, TTX-resistant sodium channels, the TRPV1 channel and receptors such as bradykinin or opioid receptors. The recent emergence of transcriptomic studies identifying a far-more diverse variety of nociceptive subtypes within dorsal root ganglia, has not only challenged the traditional classification of sensory neurons within the DRG, but the open-source nature of the generated databases provide a focal point of comparison to better classify and understand the phenotypic differences of existing DRG-derived cell lines. An important consideration regulating the phenotype of cell lines not often discussed is that a variety of different media for maintenance and differentiation of the cell lines have been used which can make it difficult to compare experiments between different groups using the same cell line but different media formulations (Table 1). Evidence from other sensory cell lines indicates that the use of different media for the culture of the same cell line can produce significant variability in the expression of a number of enzymes and transporters including the catecholaminergic biosynthetic enzymes tyrosine hydroxylase (TH) (Dixon et al., 2005). Studies analyzing the effect of media on the expression of critical neuronal or nociceptive properties are yet to be carried out on existing DRG- cell lines. Another significant aspect that has emerged from the studies reviewed here, is that a large proportion of publications do not directly refer to the overall cell population heterogeneity, which makes it challenging to draw conclusions from selected single cell measurements such as patch clamping or Ca^2+^-imaging.

A rather underappreciated and unexplored aspect of these studies is the characterization and use of differentiated cells. These immortalized cell lines are often reported with results stemming from the use of undifferentiated or spontaneously differentiated cells, which begs the question of whether many of the published studies fully utilize the potential of these cell lines to represent sensory neurons. It is evident that all of the available cell lines have the capacity to alter their characteristic features such as peptide content or excitability upon exposure to chemical differentiating agents or growth factors. Hence, the absence of RNA expression profile studies or comprehensive proteomic analyses does not allow a detailed comparison of the impact of differentiation on the neuronal or nociceptive properties of these cell lines. Although none of the cell lines reviewed here is identical to, for example, a certain nociceptor subtype, all of them show mixed expression profiles specific for nociceptors. Therefore, better characterisation and prudent differentiation with appropriate growth factor and mitotic agents could add valuable tools complementing primary cell cultures and aid in reducing the number of animals used in future studies. Besides, these refinements would further help to establish these cell lines as more valuable tools for approaches such as high throughput drug screening where primary cultures are not a viable option. Finally, immortalized cell lines from rat and mice are valuable tools in many ways but the best model for human sensory neurons would be human immortalized cells. Unfortunately, the only human DRG-derived cell line HD10.6 is no longer available to the general research community indicating an unmet need for the creation of human DRG cell lines.

### Future Prospects

All of the reviewed cell lines have characteristics that make them highly suitable for future studies. F-11 and ND23/7 cells are suitable for functional studies as the cells share strong characteristics with nociceptors upon differentiation. The presence and expression of different nociceptor-related Trp channel proteins including TRPV1 in addition to activation of ion-channels-induced release of neuropeptides suggest that differentiated F-11 cells possess a high suitability for investigations into nociceptor signaling. Testing of molecules to determine their potential to directly activate or sensitize nociceptors is crucial in developing drugs and treatments. The cells lines can be grown on different surfaces which supports the inclusion into high-throughput systems and the presence of VGCCs allows direct investigations of responses via imaging.

Recent advances in the development of calcium indicators such as GCaMP6s (Qian et al., 2019) allow the creation of stably transfected F-11 or ND7/23 cells to investigate cellular responses in characterized single populations of nociceptor-like neurons via in high throughput assays. Of importance would be human DRG cell lines such as HD10.6 for example for the prediction of nociceptive responses to anti-cancer drugs (Niu and Wang, 2015).

But the potential applications of nociceptor-like cell lines is not restricted to drug testing. There are essential questions in the area of basic science that could be addressed using the cell lines. Sensory nociceptive neurons interact with surrounding cells in the peripheral tissues and in the spinal cord by many mechanisms including via communication via exosomes. Studies investigating the generation of exosomes and sorting mechanisms of exosomes in nociceptive neurons require a homogenous population of cells. F-11 cells have already been used to investigate exosomes (Oh et al., 2017) but the mixed genetic background might restrict experimental options. 50B11 cells or MED17.11 could be used to investigate transcriptional and intercellular basic mechanisms of exosome genesis. More importantly, exosomes may serve as novel potential diagnostic biomarkers for disease conditions and provide potential therapeutic compound leads.

Pain and the development of chronic pain involves an epigenetic component. Epigenetic mechanisms are reversible which makes them attractive targets for treatment of chronic pain. But epigenetic mechanisms in nociceptors are difficult to investigate and far from clearly understood (Penas and Navarro, 2018). Both 50B11 and MED17.11 cell lines have characteristics suitable to investigate the basic mechanisms underlying epigenetic control of nociceptors and to test treatment strategies.

Of course the future advances in nociceptor neuroscience gained by using the immortalized cell lines must be closely coupled to translational experiments involving naïve neurons and whole organisms.

### The Limitations of Immortalized Cell Lines

This review describes stable immortalized cell lines that mirror nociceptive sensory neurons. These sensory neuronal-like cell lines were primarily established by fusion of post-mitotic embryonic [F-11 cell line (Platika et al., 1985a)] or neonatal [ND cell lines (Wood et al., 1990)]), rat DRG neurons with mouse N18Tg2 neuroblastoma cells, with the 50B11 (Chen et al., 2007) cells generated without inclusion of mouse genome, the MED17.11 cell line being generated from the immortomouse and the no longer available HD10.6 cell from human embryonic tissues using retroviral infection. All of these hybrid cell lines exhibit multiple DRG-selective properties, including cytoskeletal proteins, synaptic proteins, ion channels, neurotransmitters, and neurotransmitter receptors. However, the expression of markers such as tyrosine hydroxylase and neuropeptide Y in rat DRG-derived ND sub-clones (Suburo et al., 1992), but not 50B11 cells (Chen et al., 2007) confirms that cell lines are derived from different types of nociceptive neurons or that certain properties exhibited by these clones were derived from the neuroblastoma parent cell. More importantly, some of these hybrid cell lines do not contain neuropeptides such as CGRP and Substance P (Suburo et al., 1992) normally present within DRG sensory nociceptor and mechanoceptor populations. Previous reports also show that low-threshold calcium currents are the predominant component of calcium current in differentiated hybrid cells (Boland and Dingledine, 1990b; Kobrinsky et al., 1994) when compared to DRG neurons. Even though capsaicin- and bradykinin-activated currents were expressed by a subset of F-11 cells at early passages, the ability of these cells to express these properties after multiple passages appears to diminish (Kusano and Gainer, 1993). Examining the properties of retrovirally generated HD10.6 neurons, when differentiated, these cells in addition to other sensory neuronal-like cell lines did not express TTX-resistant sodium currents (Raymon et al., 1999), suggesting that some of the nociceptive properties and characteristics are independently controlled by extrinsic cues.

However, these neurons are functional and exhibit many properties specific to subsets of sensory neurons, including characteristics unique to nociceptive sensory neurons. All of these immortalized DRG-derived cell lines have the potential value for future studies of developmental neurobiology, the identification and validation of novel drug targets, and the development and implementation of drug assays targeting specific nociceptive characteristics.

In summary, immortalized DRG cell lines have the potential to develop into tools more valuable than their current utilization would indicate, and in many ways remain underutilized and incompletely characterized. However, with advanced approaches to cell differentiation and more detailed analysis of nociceptive characteristics they possess considerable potential to support research in areas such preclinical research in basic neurobiology as well as drug development and screening targeted and nociceptors.

## Author Contributions

All authors listed have made a substantial, direct and intellectual contribution to the work, and approved it for publication. RH and DM researched and summarized the publications. RH created tables. RH, DM, and CB wrote the manuscript.

## Conflict of Interest

The authors declare that the research was conducted in the absence of any commercial or financial relationships that could be construed as a potential conflict of interest.
